# Braving the Elements: Ambivalence as Opportunities for Change in Individual Psychotherapy With Men Using Intimate Partner Violence

**DOI:** 10.3389/fpsyg.2019.01693

**Published:** 2019-07-17

**Authors:** Mari Todd-Kvam, Bente Lømo, Odd Arne Tjersland

**Affiliations:** ^1^Norwegian Centre for Violence and Traumatic Stress Studies, Oslo, Norway; ^2^Department of Psychology, Faculty of Social Sciences, University of Oslo, Oslo, Norway

**Keywords:** psychotherapy research, ambivalence, intimate partner violence, domestic violence, psychotherapy process, motivation, change process, gender analysis

## Abstract

This study examines client utterances that can be understood as ambivalent in violence-focused therapy. The purpose is to enrich our scientific understanding of client contributions to therapy when they appear ambivalent to the therapeutic project and develop clinically relevant perspectives that enable us to help this and other client groups. Using constructivist grounded theory analysis of five completed therapies, we describe three categories of client ambivalence present throughout all five therapies: I am bad, but I am not that bad; I have tried and tried in vain; and I know it is wrong, but I have to, I have no choice. The categories are described and understood from a clinical perspective. They are developed on the basis of an interpretation of what seems to be at stake for the client in the here-and-now of therapy. Clinical implications are discussed.

## Introduction

*“Those who experience domestic violence can’t close the door on the world outside and feel safe”* (Det kongelige Justis- og Det kongelige Justis- og beredskapsdepartement, 2013, p. 11, author’s translation).

This study explores client utterances in violence-focused therapy, that can be understood as ambivalent toward the change project in various ways. Violence-focused therapy is a setting in which the client and therapist often struggle to establish and maintain a strong and fruitful working alliance, with high rates of drop out (Daly and Pelowski, 2000), and modest or equivocal outcomes (Smedslund et al., 2007; Eckhardt et al., 2013; Koehler et al., 2013). The analysis seeks to enrich our understanding (Stiles, 2015) of how the clients participate in this therapeutic process. We aim to build clinically relevant knowledge (McLeod, 2013; Stiles, 2013) by providing descriptions and clinical reflections that can inspire clinicians to find constructive ways of meeting clients who are experienced as ambivalent or contradictory in the therapeutic project. The purpose is first to sensitize therapists to such ambivalent utterances expressed in the therapeutic context, and second to aid reflection on how to respond in order to strengthen the alliance and encourage the clients to continue the process. The findings can also shed light on the topic of ambivalence in psychotherapy in general. It thus adds to the existing psychotherapy research literature on both ambivalence and violence-focused therapy. The remainder of this section will briefly review literature on ambivalence and on violence-focused therapy before describing in more detail our contribution to these fields and our theoretical stance.

Whilst a review of the extant general psychotherapy literature suggests that ambivalence is a frequently encountered and studied phenomenon, there is little consensus on how to define and understand it. A broadly shared understanding describes oscillations between more or less conscious contradicting motives, arguments, needs, voices or parts of the self, but ideas vary regarding what these oscillations represent, how deliberate they are, and how they relate to change. Some perspectives explore client ambivalence as a stage of deliberation early in the change process that has implications for alliance-formation and overall outcome (Prochaska and DiClemente, 1984; Taft et al., 2004; Principe et al., 2006; Miller and Rose, 2015). This perspective tends to conceptualize ambivalence as a hindrance to overcome (Moyers and Rollnick, 2002). Others examine it as a more or less deliberate part of the continuous change process, which opens up for viewing ambivalence both as a hindrance to work with throughout therapy (Gonçalves et al., 2011; Ribeiro et al., 2014) and as reflecting the inherent uncertainty in change work, and thus a potential opening for change (Shaffer and Simoneau, 2001; Abbey and Valsiner, 2005; Engle and Arkowitz, 2008; Ribeiro and Gonçalves, 2010). We will review our findings in light of these perspectives in our discussion.

Violence-focused therapy is characterized by unusual conditions that make ambivalence a useful focus. First, the clients are most often mandated to be present by court. In our study, we look at clients who choose to be in therapy without legal obligation, but nevertheless do so in a context of pressure from external agents such as partners or social services. Thus, the client might experience the goal of therapy – to stop the violence – as more or less imposed. Second, it is widely recognized that IPV has severe consequences for the physical and mental health of those exposed (Jack et al., 1995; Coker et al., 2002; Ehrensaft et al., 2003; Överlien and Hydén, 2009). The perpetration of it is equally widely viewed as unacceptable, including by the therapist and, in most cases, the client. Research on client experience finds that they often feel shame and guilt upon realizing the impact of their violence and when seeking help for it (Dutton et al., 1995; Dutton, 2007; Flinck and Paavilainen, 2008). They often express a sense of powerlessness as at the core of their use of violence (Isdal, 2000). Although we should not underestimate the courage and initiative it takes to seek therapy for this issue, we can recognize that the client’s motivation to do change work often is challenged by many other voices in the here-and now of therapy, such as protecting his sense of identity or avoiding shame and guilt. In IPV therapy, then, it might be particularly useful to understand motivation as “neither a static nor a uni-dimensional construct” (Shaffer and Simoneau, 2001, p. 100), and to examine ambivalence in the context of how conflicting voices are expressed in the therapeutic project.

The clients in violence-focused therapy have been described as using neutralizing strategies when disclosing their violence, such as denying, downplaying and externalizing the responsibility for it (Cavanagh et al., 2001; Isdal and Råkil, 2002; Flinck and Paavilainen, 2008; Catlett et al., 2010). However, two recent studies describe the behavior of these clients from a different perspective. Lømo et al. (2016) emphasize how clients make invitations to a working alliance that can be experienced as stronger or weaker by the therapist. Ørvik and Kvam (2015) and Todd-Kvam and Ørvik (unpublished) describe how clients use language in ways that both seem to foster and hinder further therapeutically productive dialogue (Bohart, 2002). For instance, they might present the violence as a personal problem or an uncontrollable event. Although use of neutralizing strategies has been repeatedly identified across different populations of men perpetrating IPV, these recent studies bring forth nuance and shed light on how the client can oscillate between contributing to the therapy process in more and less constructive ways.

We wish to further explore the way these clients contribute to therapy. However, our object of research is not the clients’ attitudes or relational function, using therapy as one of several contexts in which it emerges. Rather, it is the therapy *process*, which we aim to shed light on through looking at client utterances. We work from the understanding that in psychotherapy, the client does not struggle alone, but in dialogue with someone who is immediately present and takes part in his struggle. The client brings a self-experience to therapy consisting of multiple voices representing different motives, experiences of self and others, and needs. Telling the story about himself and his violence *in* therapy *to* the therapist affects which voices get to speak and the dynamic between them. Each utterance the client makes is affected by the actual and anticipated responses from the therapist, and by his own (more or less conscious and possibly conflicting) voices and motives *in* the dialogue. In short, “the ‘what’ and ‘to whom’ affect one another reciprocally” (Leiman, 2011, p. 455). As such, the conversation between client and therapist can be seen as a stream of relational, social and strategic interactions, mediated by language in its broadest sense (Goolishan and Anderson, 1998 – see definition p. 374; Watzlawik et al., 1967; De Jong et al., 2013). Client ambivalence in therapy is, understood like this, a psychological experience in the here and now of therapy *and* a strategic and relational speech act. It reflects how an inner dynamic or conflict between the clients’ multiple voices is represented by the client as he is struggling to make meaning in his change project with the therapist. Thus, we interpret from a constructionist, dialogical perspective (Gergen, 2015b), understanding these utterances as characteristics not of the client, but *of the change work*: how the clients express themselves to the therapist in context of the therapeutic project.

We were especially concerned with the dynamic quality of many of the utterances these clients made in therapy, both regarding the violence in specific and other topics related to change. We chose the term ambivalence to describe such *utterances containing meanings that can be understood in different, often contradictory ways that open for different directions in the therapeutic conversation.*

From this understanding, we view this ambivalent form of client utterances as a part of every phase of the therapeutic process. In line with our purpose of offering clinically relevant and constructive perspectives, we seek to explore how we can understand such ambivalence as an opportunity for change, in light of a positive change process. On that basis, we analyze ambivalence throughout completed therapies, where the client and therapist had been able to form and maintain a strong working alliance, and where rich outcome data suggest that meaningful and significant change had occurred. We will describe utterances we understand as ambivalent and offer reflections and interpretations of what they might represent in change work and how the clinician could meet them to encourage a strong working alliance.

Our research question is:

How can we understand client utterances that appear ambivalent in therapies with men who have sought and successfully completed therapy for their use of violence in their intimate relations?

## Materials and Methods

### Methodological Framework

We wished to approach the data and the analysis without presuppositions with regards to analytical considerations such as delineating meaning unit, operationalizing ambivalence and the use of specific analytical tools. We therefore chose an explorative and emergent constructivist grounded theory analysis (Charmaz, 2013a,b), and thus adopted a social constructionist epistemology (Gergen, 2015a). This allowed us to stay close to the empirical material throughout the analysis, whilst at the same time recognizing our own subjectivity and its effect on the development of our findings. Whilst the authors were not active in the therapeutic process we observed, we have actively selected parts of it to analyze and chosen ways to describe these on the basis of our interpretations. As such, we co-create our findings in interaction with the text. Furthermore, the process of reading and making meaning of this text constitutes another process of co-creative interaction (Chandler, 2002). To provide the reader with the context of our interpretations, we have included paragraphs about the context of the study and the participants, as well as the researchers’ theoretical stance outlined above.

### The Context of the Study

The data for this analysis is from a larger naturalistic outcome and process project of the treatment given at Alternative to Violence (ATV), the ATVT project. ATV is a non-profit, non-court-mandated outpatient treatment center, providing violence-focused psychotherapy. The ATVT project is run by the Norwegian Centre for Violence and traumatic Stress Studies, in collaboration with ATV. ATV therapy is integrative, using interventions derived from different therapeutic methods and traditions to work toward changing violent behavior. The goal is to end the use of violence by gaining insight into the causes and effects of it and finding alternative strategies. Specific tasks involve disclosing and recognizing responsibility for the violence, homework assignments practicing anger management strategies, exploring how the emotions and life experiences of the client relate to the violence, exploring how it has affected those around him and finding ways to repair and apologize to his loved ones. A core assumption is understanding violence as a learned strategy for dealing with difficult emotions. The content and progress of interventions in ATV therapy is individually tailored rather than based on a manual (Raakil, 2002). For instance, trauma-focused interventions will be included if considered necessary for the client to understand and prevent his violent behavior. The non-standardized approach of ATV excludes an analysis of specific shared components across cases but allows for the study of processes in individualized therapy. The approach employs a broad definition of violence, including physical, psychological, sexual, material and economic violence, and focuses on how it affects the victims.

### Data Collection and Selection

All men seeking ATV treatment during an 18-month inclusion phase were asked to participate. Clients were excluded if they were unable to attend therapy due to a severe mental health disorder such as an on-going psychotic episode or a serious drug addiction. Participants who did not have sufficient written competence in Norwegian to fill out self-report forms were also excluded. All participants signed a written consent form, and the study was approved by the Norwegian Regional committee for medical and health research ethics (REC) for the south-east. Extensive process- and outcome measures were taken throughout the process, including audiotapes of all therapy sessions. We describe the measures used in our data selection below.

Our purpose (Morrow, 2005) in selecting our material was to ensure a dense and nuanced body of client ambivalent utterances in the context of a lasting, fruitful working alliance in individual therapy. Out of 84 men attending individual therapy, 31 completed the process. Our selection focused on completed cases with a good outcome because we wanted to study how clients expressed ambivalence to the therapist throughout therapy, connected not only to the client’s experience of needing therapy or not, but to various themes related to disclosing their perception of the violence and their role in it. Furthermore, we wanted to be able to do an in-depth analysis of multiple sessions from each therapy, and thus wanted a small, but diverse set of cases.

A completed case was defined as one in which the therapist and client agreed on termination on the grounds that they had achieved significant progress. To further select cases where we could be as sure as possible that the clients had undergone meaningful and stable change, we used multiple outcome measures. First, client and partner responses on the self-report scale VAS, a shortened version of the Violence Alcohol and Substance abuse Questionnaire (VQ) (Strandmoen et al., 2016) was used. We understood meaningful and stable change as desistance from or a substantial reduction in overall use of violence both immediately and 18 months after therapy had ended. Second, we used client and partner responses to open-ended questions on how the client had changed (or, indeed not changed). Positive outcomes here were concordant descriptions of desistance from, or a substantial reduction of, violence and of better relational functioning. To secure diversity in the sample, we chose cases involving clients with different employment statuses, ages, levels of education, cultural backgrounds and different mental health issues. This selection was based on data from a demographic interview and client responses to the Outcome Questionnaire (OQ-45, Lambert and Finch, 1999) measuring mental health issues at T1. We also included cases with different therapists. This was done to ensure a material of ambivalent utterances expressed in the context of different specific others.

This selection process resulted in five cases, ranging from 13 to 40 sessions. They were all able to achieve meaningful and stable change as defined above, and rated a consistently good working alliance as measured on the first, fifth, tenth, twentieth, thirtieth and last session (Avg. ranged from 3.92 to 6.75, the median from 5 to 7 on the Working Alliance Inventory, Horvath and Greenberg, 1989). Their therapists were mostly in accordance, though with a somewhat wider range (avg. from 3.42 to 6.63, median from 3 to 7). In addition to the first and last session, sessions from various phases of each therapy were selected based on information in client and therapist session evaluation forms, indicating that important topics such as new episodes of violence or problematic interaction patterns between the client and partner had been discussed. Between 5 and 9 sessions were selected from each case depending on therapy length, resulting in a data material of verbatim transcripts of altogether 35 sessions for analysis. To ensure anonymity, all participants’ names in this article are pseudonyms. In addition, potentially identifying information, such as the specific age of the participant or the ages of his children, is either excluded or changed.

### Participants

*The clients* shared some relevant core characteristics. They had all used physical violence against their partner, some also against their children. All the clients had also used other forms of violence than physical, such as psychological or material violence. None of the clients or partners reported use of sexual violence. Whilst it was a particular incident that led some of the clients to seek help, they had all used violence on multiple occasions. However, they varied on demographic measures and came from different life situations. Their ages ranged from 31 to 60 years of age and they had completed between zero and 14 years of education after compulsory schooling. Three were born in Norway, one in Eastern Europe and one in Western Europe. One client was married, two were cohabitating, one was separated, and one was living alone at the start of therapy, but in the latter case the partner moved back in during the course of the therapy. Three lived with children. Four were employed and one was searching for employment. Some of the clients had problematic use of alcohol and marihuana. Some had used violence against people outside their intimate relations. With regard to mental health, the range of score on the OQ-45 at the start of therapy was 43 to 99 (with 4 out of 5 clients above cut off). Some of the clients reported a severe level of traumatic experiences as measured by Traumatic Experience Checklist (Nijenhuis et al., 2002), others low.

*There were four therapists*, of both genders (one was represented in two cases). They had between 3 and 16 years of clinical experience and between 2 and 15 years of experience working with this client group. In line with the integrative, individually tailored approach, the therapists used different interventions from behavioral, cognitive, dynamic and attachment approaches.

### Data Analysis

The first author listened to the audiotapes to immerse in the material. However, we were interested in exploring verbal speech acts as a relational, co-constructive and strategic behavior in therapy. Furthermore, whilst audiotapes can provide several cues regarding non-verbal aspects of communication, we assessed that video-recordings would be a better material for exploring non-verbal communication. Thus, we chose to focus on the verbal language.

In the first step, the first author read through three cases using ambivalence as a sensitizing concept (Charmaz, 2013a).

In delineating our unit of analysis, we used the term *ambivalent utterance* in line with our understanding given above: as *utterances containing meanings that can be understood in different, often contradictory ways that open for different directions in the therapeutic conversation.* Our meaning unit was the ambivalent utterance *as a whole*, encompassing the different and often contradictory meanings contained therein. This meaning unit was developed as the most meaningful way to describe and share what we observed in the text and our interpretations of it. The utterances were found within one speech act or a short segment of dialogue. We selected utterances where the different meanings contained in the utterance were expressed closely in time. This is because it is exactly the *dynamic* of this phenomenon we wished to capture.

Importantly, the interpretation in this analysis was of the linguistic actions – when the client made utterances in which one meaning could be interpreted as attenuating or conflicting with another meaning. It is not an interpretation of the client’s *intention* to attenuate. As mentioned in the introduction, the aim was to shed light on ambivalence in client utterances as an aspect of the here-and-now change work of therapy. The utterances were marked as meaning units to compare and contrast with each other. To get to the appropriate level of meaning we asked questions that were part experiential, part interpretative (i.e., clinical) (Kvale, 1989). Such questions were for instance “what is the client doing and reflecting with expressing this ambivalence to the therapist?” and “what seems to be at stake for the client when expressing this to the therapist?” Codes based on this understanding were assigned and developed in collaboration between the authors.

In the second step of analysis, two new cases were added in order to compare and contrast new data with our preliminary codes. This provided nuance and depth to our analysis but added no new main codes.

In a third step, different ways of conceptualizing similarities and differences between the codes were discussed. The first author suggested conceptualizations and categories and presented examples. The three authors met bi-weekly to monthly to discuss and reach consensus. We developed three main categories and a set of subcategories based on an interpretation of what seemed to be at stake for the client in the here-and now of therapy.

## Findings

**FIGURE 1:**
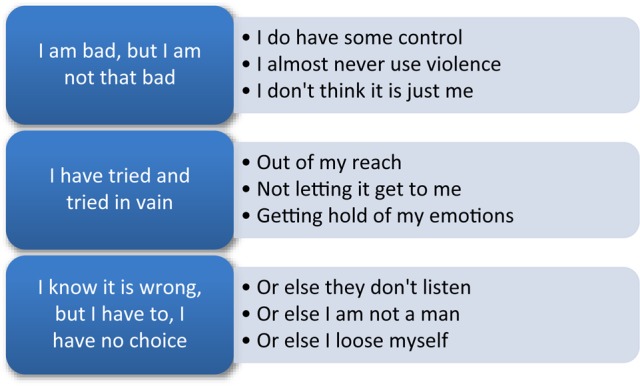
Main- and subcategories.

In the three categories (see Figure 1), the different meanings contained in the ambivalent utterances, as expected, rarely concerned whether or not the client wanted to stop using violence – his *decision to change*. The ambivalence seemed instead to be focused on the nature of his violence or behaviors related to it, such as both emphasizing and downplaying the severity of the violence within one utterance. We have chosen to describe the categories with a language that is close to the clients’ voices. The purpose of this is to represent them in a way that reflects what clinicians might meet in their own practice and orient the reader toward the perspective of the client’s experience. All three categories of ambivalent utterances feature throughout the therapies in all five clients, though with differences in respect of when they first occurred and how they were distributed throughout the therapy. This is described in more detail under each main category.

### I Am Bad, but I Am Not That Bad

In this set of ambivalent utterances, we observed a dynamic between presenting the use of violence and attenuating the significance of it. On the one hand, the clients describe their violent acts, their role in the interaction leading up to the violence, or the consequences the violence has had on their loved ones. On the other, they emphasize how rarely it happens, how short-lasting it is or how they are not the only one responsible. What these ambivalent utterances have in common is that they happen in a therapeutic context of shame and stigma (being someone who uses violence against his loved ones). In one example the client even directly appeals to the therapist: “don’t get me wrong, it was just ….” The attenuating meaning often describes what they *do* achieve (only using violence very rarely, not hitting) or on what they are *not* (the kind of guy who chokes his son intentionally). This leaves us with the impression that it is important to the client both that he recognizes the severity of his actions to the therapist, but also that he makes sure the therapist sees the boundaries of his violence. What seems to be at stake for the client is how the therapist sees him as a person.

#### I Do Have Some Control

One way of presenting the violence as a problem and at the same time protecting his self-representation was by contrasting himself and his violent actions to more severe violent actions or to a more hostile person.

In one example, the client describes an episode of violence toward his son. He oscillates between recognizing the severity and consequences of his violence and focusing on how it was not very hard and not intentional. It seems like it is important to the client to make sure that the therapist does not perceive him as a worse person than he is: someone who chokes his son hard and who does so intentionally:

“T: so, you held him by the throat – or?

C: Yes, but it wasn’t hard really. He says I choked him, but really, I was just trying to straighten him up, you know, stand still when I am talking to you. So, it wasn’t like that, it was just like pushing him, sort of.

T: Yes, but up here?

C: Yes.

T: That feels very...

C: Yeah, yeah, he felt it. And he remembers it.

T: Yes. How old was he then?

C: He is twelve now. Maybe ten? Nine or ten.

T: Yes.

*C:* Please don’t get me wrong, it wasn’t like that, it was just: oy!”

Frank, session 1

In another example, the client brings up the episode that brought him to seek therapy. He came home intoxicated and became verbally aggressive toward his partner. When she tried to escape the situation, he physically held her back, leading to his partner’s son getting injured while trying to break them apart and their son (a toddler) waking up in distress.

*“Uh, there was an episode where I, uh... uh... simply crossed the line, and, uh, I didn’t hit her or anything like that, but it uh, it turned into a scuffle that uh, with children there, her children, uh... and uh, it was something that absolutely, absolutely should not have happened.*”

Andrew, session 1

In his description, the client emphasizes the severity of his violence (I crossed the line) while, at the same time, ensuring that the therapist does not get the impression he *hit* his partner. He attenuates the severity of his actions by describing more severe acts which he did not commit. He then returns to the emphasis on severity: It really, really shouldn’t have happened. It is as if the client is concerned with making sure that the therapist knows both that he takes the violence seriously, but also that he was able to avoid using more severe violence.

#### I Almost Never Use Violence

Another way of presenting violence as a task for therapy and at the same time represent himself as a decent person was by focusing on both the violence and on the rarity of it – on all the times he is able to *not* use violence.

In one example, the therapist explores how the violence plays out in the everyday life of the client and his family. The client describes an explosive temperament and verbal aggression, and the therapist enquire into what triggers this:

“C: it can be anything

T: It can be anything

C: yeah, yeah.

T: yes

*C: But it’s not like I do it every day or every week or anything like that, but it builds up inside of me.*”

Frank, session 1

The client describes himself as easily triggered. When the therapist affirms this, it is as if the client becomes aware of the light this puts him in and seeks to rectify that by emphasizing the rarity of it: he is able to refrain from using violence most days.

In another example, the client highlights the fact that the violence now happens less often. It is as if upon recognizing that it is still a problem, the client feels a need to emphasize to the therapist what he *has* achieved (reducing the frequency of his outbursts).

Client: “Both yes and no, basically (uncertain), I have had a couple uh, a couple of uh, outbursts, it, it has improved slightly, I am working on it all the time. (t: mhm) So, so I’ve had a couple episodes, (t: mhm) I think. (t: mhm) And... yeah. I have an okay feeling about it. (t: mhm) It, it, it isn’t getting worse and worse. That was kind of (laughs) what was the problem this spring and summer anyway…”

Luke, session 3

#### I Don’t Think It’s Just Me

The final variation we observed in this category was utterances in which the ambivalence the client expressed revolved around whether he had sole responsibility for the violence and for its impact. On the one hand, the clients focus on and recognize their responsibility, but on the other, they downplay it. It seems as if accepting responsibility to the therapist makes them simultaneously want to protect their own self-representation by emphasizing the other person’s contribution in the violent episode or in how the consequences play out in the aftermath.

In one example, the client brings up the possibility that he contributes to the relationship dynamic that often ends up in him using violence, but then immediately emphasizes that his partner’s accusations are exaggerated:

*“Well, well, uh, then you start to think that uh, perhaps I ought to be more attentive to her, and, and (short pause). Well, well, what she is saying is an exaggeration anyways.*”

Phillip, session 2

It is as if he wants to address his behavior but feels that he must make sure that the therapist does not come to share his wife’s perspective: that he is as horrible as she claims.

In another example, the utterance contains several equivocations between the client on the one hand reflecting on how his violence has affected his son, and on the other doubting that it really could have affected him so severely:

*“But something I’ve also thought about is that... eh, is that young children, they react to, to stress. They are very... they pick it up very easily if the parents are stressed or angry or something like that. So they let it, well it, it affects them (t: mhm) so much that it often affects their stomachs. (t:mhm) Um, but as much as I try I can’t see that he would have been so affected that it would upset his stomach, because it hasn’t been that bad. (t:mhm) and that one episode, the reason we’re sitting here now... (T: mhm) he slept (t:mhm). Of course, it was a stressful situation when he woke up. (t:mhm) But a situation like that should not be enough to, I mean, to damage his entire digestive system, I don’t think. (t:mhm) But when we’re talking a little, like, loudly and things like that, (t:mhm) then he screams. (t:mhm) Like (makes a screaming sound) uh, he, he reacts. (t:mhm) So if we have a uhh, we don’t need, like, don’t need to argue or anything like that, (t:mhm) but just if we speak very loudly and just, like, firmly to each other, (t:mhm) if we have a discussion at the dinner table, then, uhh, then, uuh (t:mhm) he lets us know (t:mhm) he doesn’t like it. (t:mhm) So I guess he is very sensitive to that sort of thing*…”

Andrew, session 3

This utterance serves as a good example of this article’s title. The client braves the question “have I damaged my son?,” with great trepidation and manages to bring up the topic despite the serious threat it poses to how he is seen by the therapist and how he sees himself.

Summed up, in *I am bad, but I am not that bad* the commonality is the dynamic between presenting the violence as a problem and at the same time presenting a nuance or augmentation that puts the client in a better light. We interpret this as the client’s self-representation and -esteem in the therapeutic relationship being at stake. This type of dynamic occurred throughout the therapies, but more frequently in the beginning and if the client and therapist discussed new episodes of violence.

### I Have Tried and Tried in Vain

This category describes ambivalent utterances where the client expresses a wish to end acting violently and at the same time expresses his perception that such change is unachievable (or very difficult) to achieve. They often describe a situation where they have tried to stop their violence, but where these attempts have been impossible to carry out in practice. The ambivalence is thus between describing the attempts and strategies they have used to change their violent behavior and expressing resignation and powerlessness about achieving this change. In these ambivalent utterances, the clients’ sense of agency and experience of hope seem to be at stake.

#### Out of My Reach

One variety is ambivalence between wanting to end the violence and experiencing it as something out of the client’s reach.

In one example, the client describes the relationship dynamic that often ends up in him using violence, when he experiences his wife as attacking him verbally:

*“I think that she has* …*it may be that she that she has a personality disorder of one kind or another. I am not trying to excuse myself [from taking responsibility], that’s not what I want. But, like, she is kind and loving for short periods, but then it’s, like, just stupid a statement from me, lack of consideration, that I speak loudly, that I fail in the big and the little things. Then she becomes implacable, furious, unable to see what’s behind my actions. And it never moves* …*I am never able to moderate her position, in a way*.”

Phillip, session 1

The client emphasizes to the therapist that he wants to take responsibility for changing his violent behavior toward his partner. At the same time, he represents the solution to this as changing *her* – moderating her point of view. As in the category “There’s more to it than me” below, the client points to the partner. However, whilst he is clear that the violence is not her responsibility, *his* task, *his* strategy to end the violence – changing her – is impossible.

In another example, the client describes changing as impossible because of his father:

“Actually yeah... He [the father] said to me that I have the same genes and DNA as he does. My mother tells me that when he [the father] was young he just drunk and fought all the time (...) When I was angry, I said to him that, (...) I told him, when I was angry, that ‘argh. I am your son, so, really I am your fault.’ Because I have to do what you have done, I cannot change it. Now I must change, I must see a therapist”

Alexander, session 1

The client starts by describing how his father was a bad seed, and how this determines his choices for him, including the violence. In this example, it is not the solution (seeing a therapist) that is experienced as impossible, but the conditions for changing his violence: having his father’s “bad DNA.”

#### Not Letting It Get to Me – Remaining Unaffected by the Emotions of Others or One’s Own Emotions

Another variety of this dynamic is oscillations between, on the one hand, expressing motivation to stop using violence and, on the other, describing an unattainable solution to this as somehow *not being affected* – either by one’s own emotions or the emotions of others. In these examples, the focus is both on factors outside and within the clients: the partner and children, the anger. However, the solution is not represented as changing these factors. Rather it is represented as not being affected by them or as restraining the effect they have.

In one example, the client describes the dynamic he experiences as contributing to his use of violence. He alternates between pointing to how the partner needs to change and emphasizing that he is responsible for controlling himself:

*“Uhhh, and it is very easy, it is very easy to fall into that trap that uh, to blame others. (t:mhm) That it is* ***your*** *fault, (t:mhm) that* ***you*** *have to be there for*, ***for me***. *(t:yes) So that I can get better. (t:mhm) Uh, but that, that’s not how it is, really. (t:mhm) You have to take control uh, of yourself, if you see what I mean. (t:mhm) Uh, and handle it accordingly, but, uh, there’s no getting around that, like, you live in a, you live in a relationship, and, uhhh, you live together, like, everything around you affects you.*”

Andrew, session 3

Again, the client refers to his partner, but this time describes the solution not as changing her, but as being able to remain unaffected by her. At the same time, he describes this solution as difficult to achieve; there is no getting around being affected.

In another example, it is the restraint of anger that is described as the impossible solution:

*“Then he [the son] started misbehaving. And I had a log in my hands, ok? (t:mhm) And so, when I heard the noise, I wanted to throw that log (T:mhm). But I didn’t (t: no). But I got really… I felt it rising up inside me.*”

Frank, session 2

In this utterance, the client describes an example of having used restraint to avoid violence. However, he notes that the problem (the anger) was still there inside of him. That is, his impossible task is not to avoid getting angry with his loved ones, but to be able to restrain this anger. In an utterance further on, he expresses how he experiences this restraint as an impossible solution in the longer run:

*“Yeah, I do try [to avoid violence by restraining his anger], but you don’t know for how long it’ll work, you know*”

Frank, session 2

#### Getting Hold of My Emotions

This last variety is ambivalence where the dynamic is between presenting the solution to stop the violence as getting in touch with and dealing with the emotions that cause it, yet experiencing this as a hopeless and futile endeavor. Whereas the previous category described experiencing the emotions, but struggling to restrain them, this category describes difficulties getting access to the emotions – experiencing them at all.

*“I find it, it is a slow (uncertain) and difficult process, I don’t think that I’ve come all that far, really, (t:mhm) myself. So maybe I understand a lot of stuff intellectually, but (t:mhm) from there to actually getting (t:mhm) getting organized and sorted out in my feelings, and (t:mhm (clears throat)), what can I say, to being normal (laughs a little) uh, that sure isn’t easy, it isn’t (short laugh)*”

Luke, session 1

The client describes having tried for a long time to get to what it is inside of him that causes the violence, to sort this out and become normal. At the same time, he describes this process as one he does not know how to handle.

Summed up, these utterances describe how the clients experience having *tried and tried in vain*. The strategy that each of the different clients present as the solution they tried ranged from the external focus of changing the partner to the internal foci of either not being affected by others or one’s own emotions, or exploring the emotions that cause the violence. The similarity is that, whilst they all focus on their own responsibility to end the violence, they also describe their chosen solution as impossible to achieve. This dynamic occurred throughout all five therapies.

### I Know It Is Wrong, but I Have to, I Have No Choice

This category describes ambivalent utterances in which the client alternates between recognizing the need to change a behavior, such as his violence or his form of emotional communication, and underlining the necessity of this behavior, because of its effect. The nature of the effect varies. As in the previous category, there is an element of determinism in these utterances. However, whereas the second category describes the experience of trying and failing – of powerlessness or resignation, this category describes the experience of *unquestioned necessity*: *having to* use violence, *having to* hold back on displaying emotions or vulnerability, *having to* protect oneself in the reconciliation process. The specific effects detailed and the unquestioned and often insistent way they are described leads us to interpret that something at the core of the clients’ sense of self and identity is at stake. When occurring early in therapy, the effect was often used as an explanation, with little elaboration as to why this effect was necessary or whether it was possible to achieve in an alternative way. Examples of this ambivalence from later in the therapies tended to be more elaborate, including reflections on how changing previous behaviors could challenge their sense of identity. Across the category, the clients’ ideas of what it means to be a man, of masculine identity, seems to play a part.

#### Or Else They Don’t Listen

One dynamic was between the desire to not use violence and the imperative of maintaining respect and authority: to be heard and not opposed.

In one example, the therapist explores how the client’s frequent verbal aggression affects his family:

“T: Do you think they [the children] kind of walk on eggshells to avoid making you angry?

C: My wife says so

T: yeah.

*C: But I don’t think it is, not every day, I don’t think. But they tell me it is so, not every day like, but every now and then you know. When you talk to them and talk to them and talk to them time and again and they don’t react, but when I raise my voice they do react.*”

Frank, session 1

The client both recognizes the effect of the violence on his loved ones (they walk on eggshells) and focuses on how this verbal aggression and threat *must* happen: when he raises his voice, they *do* react. It is as if being heard and having authority is *necessary*, and the violence is an unwanted but unavoidable step to achieve this. The client does not elaborate on why being heard is so crucial to him.

In another example, the client describes an episode of violence. He experienced his partner as criticizing him and pushed her while she was holding their toddler. His focus is on why he had to do it:

*“Uuh …so I back off a few meters and then, ehm (pause) apologize, that it is no, uuh... yeah. Uuh (pause). The essence of what I say to her afterwards is that I don’t have the nerves to deal with that sort of behavior from her and …you just have to... (pause).*”

Luke, session 3

Whilst disclosing the violence, the client concludes by emphasizing the necessity of it in order to make the partner stop criticizing him. Again, the client both admits the violence to the therapist and describes an experience of not having a choice, whilst not elaborating on why his partner’s criticism was intolerable to him.

#### Or Else I Am Not a Man

Another variety of this dynamic was utterances that alternated between describing the experience of being vulnerable and describing the need to protect one’s sense of masculinity. It is as if the two are experienced as mutually exclusive.

In one example, the client reflects upon his learning to talk about his emotions as part of his change process:

*“Yeah, I’ve come a long way, if you see what I mean... But... if you look at it that way (laughs), if you were to use... In being a well-functioning man in today’s society, I have come a long way, for sure, so it is really just nitpicking the last bit. But I am working to try to get even a little bit further, if you see what I mean. I really am. The risk...or...not risk, but...deep within I guess there is a kind of... ingrained fear that I might (hesitant) come across as...ehm...feminine...(T:mhm)... Like we have talked about, th... so that’s…* ”

Andrew, session 29

In this utterance, the client describes his progress regarding emotional expressivity and talk – that he is now far better at talking about emotions with his partner. He then hesitantly expresses a fear that this development might pose a threat to his masculine identity (coming across as feminine), as if he experiences talking about emotions with one’s partner as an emasculating behavior.

In another example, the client starts by describing how vulnerability (crying) is interconnected to his aggression (wanting to do bad things to people) and then swiftly goes on to insist that he is not homosexual:

*“You know, I want to do bad things to people I don’t know, but five seconds later I can cry (t:mhm), and then (t:mhm) ‘oh my god, why?’ (t:yeah). I am (pause) maybe not a homosexual, (T:no), (...) I am not* ‘*gay*’.”

Alexander, session 6

The client seems puzzled by the crying. It seems that this perplexity sparks a need to clarify to the therapist that he is indeed heterosexual, as if he experiences crying as a threat to his sense of belonging to the dominant masculinity within his field.

#### Or Else I Lose Myself

A final variety is utterances where the dynamic is between bringing up something that is relevant to the client’s change process and at the same time describing this as a potential and quite fundamental threat to his very sense of existence or self.

In one example, the act of apologizing for his violent behavior is contrasted with being allowed to be who he is:

*“Then it’s back to this, that I should completely, like, throw my hands up, grovel, (t: yes), and cry over the fact that I am in therapy at all because of this (t: oh, ok). And that I have to understand that our daughter is hurt because of it, and it is clear that she thinks it’s hurtful (t: yes). But that isn’t... that doesn’t mean that I should stop* ***being me*** *(T: no) because of this (T: you shouldn’t). It rather means that I should be more clear (T: mhm) about who I am*”

Phillip, session 21

On the one hand, the client describes what he experiences as an unreasonable demand from his wife to apologize in a way that involves completely throwing his hands up and subordinating himself to her. On the other, he recognizes that his violence has had negative consequences for his family. He then goes back to emphasize that he does not wish to stop *being himself*. It is as if he experiences admitting his wrongdoings to his wife as a threat to his very existence: apologizing is surrendering and in opposition to being yourself.

Across these varieties, the clients describe an experience of having to, of *not having a choice*. What seems to make the violence or relevant behavior so unquestioningly necessary is that it protects a core part of the clients’ sense of identity as a man, partner and father. As such, the third category sheds light on how the clients’ fundamental sense of identity seems to be at stake. A gendered interpretation of the ambivalence presented in this category might be useful and will be elaborated upon in the discussion.

## Discussion

The purpose of this research was two-fold. First, to enrich our understanding of client contributions to therapy when they appear ambivalent to the therapeutic project. Second, to inspire clinical reflections as to what this ambivalence might represent and how it can be understood and met in a way that encourages the establishment and maintenance of a shared strong working alliance. The aim was to develop knowledge that is close to clinical practice and useful to the clinician in her work with this and other client groups (Miller, 2009; Rennie, 2012; Charmaz, 2013b). All five clients were able to achieve a positive change in their use of violence and relational functioning and they rated their alliance with the therapist as good throughout therapy. The client utterances were understood as reflecting an ambivalence emerging in the here-and-now of therapy, and at the same time constituting a relational and strategic speech act in interaction with the therapist. We developed three categories of ambivalence, based on the question “what seems to be at stake for the client?”: *I am bad, but I am not that bad, I have tried and tried in vain* and *I have to, I have no choice*. All three categories of ambivalence were present throughout all five therapies.

In the first category, the ambivalent utterances revolved around admitting to having done something bad and attenuating this with a focus on something that seemed to make it less bad. This category showed how much is at stake for the client when disclosing his violence. It seemed crucial to the client how the therapist saw him and thus how he saw himself – what sort of regard for the client the two of them were able to co-create.

In the second category, the client described to the therapist the strategies he had tried to use to end the violence, but at the same time expressed resignation and hopelessness. This category illuminates the powerlessness these clients seem to experience in their relationships and their struggles against their own use of violence. It might be important to be attentive to this powerlessness, as it is in stark contrast to how men perpetrating intimate partner violence are often perceived.

In the final category, the clients simultaneously emphasized the unacceptability of the violence and its necessity. They described their reasons for problematic behaviors (such as violence or avoiding emotional communication) in a way that made us understand it as profoundly important for them – as if the effect of these behaviors was to protect their very sense of self. This category of ambivalence predominantly occurred late in the therapies. Some of the clients expressed it early on, but these expressions were very thin in their descriptions of the effect or of what made the effect necessary. One might understand this pattern as indicating that discussing what drives them to use violence or behave in ways that contribute to it is particularly vulnerable – that it requires some time for building an alliance with the therapist and getting more comfortable with the challenging and strenuous work of introspection.

**FIGURE 2:**
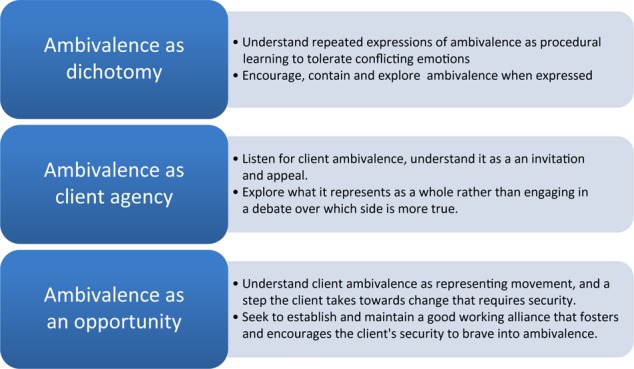
Main discussion points and clinical implications.

In the following, we discuss two further interpretations from our understanding of client ambivalence as an aspect of the therapy process. We reflect on some clinical implications and sum up by reflecting on how this analysis sheds light on ambivalence as an opportunity for change. Figure 2 sums up our main interpretations of our findings, and possible clinical implications.

### Ambivalence as a Dichotomy

The ambivalence the client expressed in therapy often seemed to rest on an assumption of a dichotomy, of mutual exclusiveness. Such assumptions, based on the clients’ experienced and anticipated interactions with the therapists (and others before them), set the stage for the dynamic between the different voices the clients express in this context. As such, they are an important conditioning factor for the process. All three categories highlight a set of such assumptions. In *I am bad, but I am not that bad*, one can interpret experienced assumptions like “either you are a good person, *or* you are a person that uses violence” or “either it is entirely my fault or entirely hers.” It is as if it is not possible for the client when approaching this topic in therapy to be both good and bad, or at fault for one’s own actions even when one experiences the other person as contributing too. Thus, the voice in the client that wants to disclose and recognize the violence in therapy comes to be in conflict with the voice that speaks of his need to see himself and to be seen as a good person. This seemed to make it difficult for the client to disclose the violence or start discussing his part in it, which we argue is fundamental for this change work. In *I have tried and tried in vain*, one can imagine an assumption of “either I succeeded in stopping violence with this strategy, or I am powerless to change.” It is as if it is experienced as impossible for the client in therapy to both recognize the violence as a problem and at the same time recognize that there are possible solutions to it. This might make it a challenge for the client to address his agency and responsibility in choosing to use violence, and thus the option of choosing differently. In *I have to, I have no choice*, one can imagine a less elaborated assumption of “either I achieve this, or…,” for instance “either my children and partner listen to me, or….” It is as if the idea of *not* achieving the effect (i.e., being masculine in a certain way, being listened to) is fundamentally threatening to the client, so much so that the rest of the sentence (or what) remains unspoken both to the therapist and to himself. In dialogue with the therapist, the voice embodying the shame and the threat to his self-image that having children who don’t listen or being a man who talks about emotions represents seems to come into conflict with- and almost outcompete- the voice representing a desire to change. This seemed to hinder the client from reflecting on achieving the effect in a different way, or indeed questioning whether and why he needs that effect in the first place. The client experiencing the effect of the violence (for instance as a way to defend oneself or vent one’s feelings) as important and as a hindrance for desisting from violence has also been described elsewhere (Flinck and Paavilainen, 2008).

This form of mutual exclusiveness resembles what has been described as “dichotomizing” (Valsiner and Abbey, 2006; Ribeiro and Gonçalves, 2010), and seems to be a hindrance to the client in changing his violent behavior. It is as if any voice in the client making an effort to move away from the violence by addressing it as a personal problem or reflecting on his own agency in changing it is barred by a different voice: the one protecting the client’s self-regard or the one speaking about his experience of powerlessness. In such a dynamic “the Dialogical Self is trapped in this cyclical relation, making ambivalence impossible to overcome within this form itself.” (Ribeiro and Gonçalves, 2010, p. 122).

These assumptions can also be understood as representing an implicative dilemma in the therapeutic process, as described by Feixas and Saúl (2005). They suggest that change in one domain (such as violent behavior) is inhibited by being associated with change in a domain the client does not wish to change (such as being a man, being seen as a good person). As stated in Feixas and Saúl (2005, p. 138), Ryle (1979) describes concisely how such dilemmas are implicative:

“Dilemmas can be expressed in the form of ‘either/or’ (false dichotomies that restrict the range of choice), or of ‘if/then’ (false assumptions of association that similarly inhibit change). Two common dilemmas could be expressed as follows: (1) ‘in relationships I am *either* close to someone and feel smothered, or I am cut off and feel lonely’; (…) (2) ‘I feel that *if* I am masculine *then* I have to be insensitive’” (italics in the original).

There are some useful clinical implications that can be drawn from understanding ambivalent utterances as patterns of thinking and talking that form an experience of an unsolvable dilemma in the therapeutic project. One hypothesis is that the force keeping the client in these unsolved ambivalences is conflicting emotions in the therapeutic space that are difficult to tolerate, such as anxiety or shame (Dutton et al., 1995; Abbey and Valsiner, 2005; Dutton, 2007; Engle and Arkowitz, 2008). If taking a step toward admitting violence, or recognizing that this violence was a choice rather than the only solution to an impossible situation, means representing oneself to the therapist as in essence an unacceptable person, then one can imagine the anxiety and threat of shame that hinders such steps. If so, one clinical application is viewing the repeated expressions of this ambivalence in therapy as an exercise in tolerating such conflict and difficult emotions. Every time the client experiences and expresses this ambivalence with the therapist, he has a chance to learn that such conflicting emotions can be tolerable. Through such procedural learning he is able to tolerate and thus experience his ambivalence (Shaffer and Simoneau, 2001), which can create space to see new nuances and different perspectives (Solbakken et al., 2011, 2012). Encouraging ambivalence, then, and both exploring and containing it when it shows up, might be a fruitful therapeutic approach (Feixas and Saúl, 2005). By repeatedly exploring these experienced dichotomies, the therapist can help the client broaden his experience of possible actions, such as new strategies to end the violence that are not impossible, or ways in which he can change some behaviors without losing his sense of masculine identity. It can also create space for the client and therapist to understand more about why these dichotomies have become deadlocked to begin with, and what is at stake for the client when he expresses them. With time, then, it might be possible to be *both* good and bad, to see more fully his own experience *and* hers, to be a good father *without* being authoritarian and to be *both* vulnerable and masculine.

### Ambivalence as Client Agency

Seeing ambivalent utterances as potential starting points for change provides some perspectives that can be clinically useful. As noted in the introduction, we might assume that when the client expresses himself in ways that come across as contradictory, he too experiences it as conflicted in the here-and-now of therapy. When he chooses to represent this conflict to the therapist, it can be understood as expression of agency in his change process, in that it constitutes a step that he takes in order to achieve change.

In the first category, “*I am bad, but I am not that bad*” the client can be understood as seeking to ensure that the therapist sees him as a good person. At the same time, he seeks the therapist’s help to resolve the conflict between the need to see himself as a good person and the desire to recognize and deal with the violence. We understand this as a step toward alliance building by securing a shared understanding of the problem (as described in Lømo et al., 2019), and by contributing to the bond that allows the client to feel secure enough to explore these shameful topics. This could explain why this category of ambivalence was more prevalent in the beginning of therapy, and when clients disclosed new episodes of violence. These are phases of constructing and reconstructing an alliance around him as a person using violence. The experience of being seen as a good and normal person as a prerequisite for daring to enter into change work resonates with findings from group therapy (Silvergleid and Mankowski, 2006; Gray et al., 2014). In one example, one of the clients explained that a positive aspect of the group was to “find the men in the group to be basically good people. And I found out that I am a good person, too.” (Gondolf and Hanneken, 1987, p. 187).

In the second category, “*I have tried and tried in vain*,” the client expresses himself as deadlocked in a way that chimes with literature on powerlessness being at the core of this client group’s experience (Isdal, 2000). However, the client *does* present this deadlock *to* the therapist. In a clinical context, these utterances can be understood as the client saying: this is where *I* end and *we* begin in my work to end this violence. Addressing one’s previous solution strategies can be understood as client engagement and an invitation to co-create agreement on the task of therapy (Lømo et al., 2019). From the therapist’s perspective, Oddli and Rønnestad (2012) describe exploration of the client’s theory of change and his past and current solution strategies as one of the techniques experienced therapists use to strengthen the client’s agency.

In the third category, “*I have to, I have no choice*” the client hesitantly addresses very vulnerable issues – I *do* want to change, but I am scared of losing my sense of identity as a father and a man. The gender perspective can illuminate how notions of relative dominance and masculinity plays out in the interactions between the client and his loved ones (Haavind, 2000). In the first subcategory, the violence seems to protect the clients’ perceptions of what it means to be a successful father and partner; having authority. In the second, the clients describe experiencing vulnerability and emotional openness as a threat to their sense of masculinity. In the third, the act of apologizing seems to be associated with showing complete submission. It is as if the client is appealing to the therapist to help him see a way he can change without losing important parts of himself; a way that he can negotiate his identity in relationship to the hegemonic masculinities within his fields (Coles, 2009). The observation that some masculinities seemed to be a hindrance for the client in implementing his change chimes with previous studies (Pandya and Gingerich, 2002; Pandya, 2009). Men who have changed away from violence also describe their change processes as in part redefining their idea of masculinity and thus broadening their repertoire of behavior (Gondolf and Hanneken, 1987).

Furthermore, this category in particular resonates with the previously mentioned descriptions of implicative dilemmas, and findings that persons who struggle with mental health issues experience more of such dilemmas (Feixas and Saúl, 2005; Feixas et al., 2014). It is as if the client brings his perceived unsolvable dilemma to the table, for them to look at together.

In sum, clinical implications of this could be to listen for these ambivalent utterances in therapy and build on them as dynamic appeals and invitations (Lømo et al., 2016) rather than seeing part of it, as neutralizing strategies or resistance. The latter may invoke hostile understandings of the client and thus encourage less constructive dynamics in the therapy (von der Lippe et al., 2008). This client agency can facilitate the therapeutic process by showing the therapist exactly what it is that he struggles with in his change process, and need to co-construct a more helpful understanding of. Both “sides” of the ambivalence carry some truth – a useful clinical application might be to explore what the dynamic as a whole represents rather that engaging in a debate over which side is more true. If the therapist can create a secure space where the client gets to explore and express his experience, it might open up for a narrative and cognitive transformation where a non-violent self is possible (Summers and Tudor, 2000; Maruna, 2001; Ribeiro and Gonçalves, 2010; Ribeiro et al., 2014).

### Reflections on Ambivalence as an Opportunity for Change

The arguments above are about how client ambivalence can be understood as an important aspect of change work. Central in this is taking in the dynamic, the *movement* it represents. In our view, the dynamic contained within for instance *I am bad, but I am not that bad*, is more conducive to change than not being ambivalent about it. Denying the violence or its negative effects is clearly not conducive to change, but a too eager embrace of the, often shameful, opposite might be hindering as well. If the shame of recognizing the violence prevents the client from conceiving it as a personal problem, the therapist-client dyad might agree un-ambivalently about the goal of the therapy (stopping violence), but struggle to arrive at an agreement concerning the tasks (Lømo et al., 2016). That is, the client might agree that the violence is bad, but may fail to explore the dynamic that causes it to happen in spite of this. This seems to characterize less fruitful client-therapist dynamics (Lømo et al., 2019). When expressing ambivalence to the therapist, the client has the courage to show him or her the conflicts and contradictions he is struggling with in his change work, that are stopping him from changing his violent behavior. As a therapist, a clinical implication is to actively work to contribute to the establishment of a safe therapeutic space where the client can feel able to express this ambivalence. Ambivalence involves leaving the safe but stable notions of the violence being her fault or a necessary part of being a good father or man, and braving the elements into the new and scary territory of “I might be (part) bad” or “I might have damaged my son.” This process inevitably involves insecurity (Abbey and Valsiner, 2005). Indeed, the emotional arousal experienced in disclosing this ambivalence is a key element of the change process (Lane et al., 2015), and, as we have argued above, this exercise in tolerating emotional activation is itself therapeutic. Expressing ambivalence represents an initiative made by the client toward co-creating new understandings that create new therapeutic opportunities. As such, ambivalence can be understood as an opportunity for change worth listening for and as a “development catalyzer” (Ribeiro and Gonçalves, 2010, p. 116). This is important, as how we interpret the client’s contributions to therapy affects how we respond (Engle and Arkowitz, 2008; von der Lippe et al., 2008).

### Strengths and Limitations

First, we chose to work from a descriptive definition of ambivalence based on what we observed and interpreted in interaction with the text, and not from any of the definitions of ambivalence that exist in the literature. This might merit the question of construct validity: is ambivalence the right term to describe these observations? There are multiple other ways of understanding these utterances. For instance, one can indeed understand the clients’ semiotic attenuations as neutralizing strategies. Another understanding of the clients’ disclosure of violence and acknowledgment of it is as a personal problem is that, in doing so, he is displaying an adequate understanding of how to perform his role as a client (Goffman, 1959). However, in our search for a suitable expression to describe our interpretation of the material, the word ambivalence – ambi valence – gave “a sense of the correctness of the fit” (Rennie, 2012, p. 389). It was the presence of multiple meanings or voices with different and often contradictory valences that caught our attention in the first place, and it is this dynamic that we wish to capture and share our understanding of with the reader by using the term ambivalence. Furthermore, based on an understanding of language as constituting reality (Gergen, 2015a,b), we argue that using the word ambivalence helps create an awareness in the therapist of the dynamic of these utterances – and the implications this has for understanding the client’s experience, agency and invitations to do change work throughout therapy. As such, we believe it is a useful adoption of the term and one that constitutes a “good” reality (Kvale, 1989, p. 76).

Second, this analysis was limited to cases with a good and stable outcome. Client ambivalence might be less relevant, or different, in therapies where the client and therapist do not manage to build a successful alliance together. For instance, ambivalence in cases where the client drops out early might revolve around whether or not he feels a need and motivation to change. In our view, this is also interesting to explore, but not what we sought to illuminate with our research question and sample. Furthermore, limiting our material to only five cases might risk a biased sample. For our purposes, we considered the benefits of the opportunity of an in-depth analysis into each therapy’s unique style and processes by looking at multiple sessions from each case to outweigh the potential risk of bias in selecting only five clients. However, we deliberately used demographic measures to ensure diversity among these five and chose cases with different therapists, to lower the risk of such bias. What we saw and chose to focus on as a shared dynamic throughout the therapies and across the different therapist-client dyads was utterances that could be understood as ambivalent. Finally, the study examines client ambivalent utterances in light of this therapeutic context, but does not specifically include the therapists’ responses, which we hope to take a closer look at in a future analysis of the material. This might exclude important insights into how we can meet this ambivalence as therapists. However, our intention in this analysis was to show the nuances and variations in how client ambivalence as a relational and strategic speech act is expressed within a context of a strong working alliance, and to encourage clinical reflections around it. The purpose was to find ways of understanding it that could help the therapist get in position to help, and thus contribute to a process of co-construction that enables change (Miller, 2009; Rennie, 2012).

Third, we examined client ambivalence in a context that is uncommon in an international perspective. Most therapies do not target IPV perpetration, and those who do are most often group based, court-mandated and, we argue, more psycho-educational than psychotherapeutic. This might limit the extent to which these findings are relevant. Our hypothesis is that client ambivalence might be a part of the change process across different contexts – both for psychotherapy in general and for the process of desisting from IPV perpetration in general. We have referred to literature supporting this hypothesis in both contexts. Examining ambivalence in the context of IPV therapy is advantageous because of both its prominence (it occurs frequently throughout the therapeutic process in our data) and its salience (it is, we argue, an important element of opening for change). Furthermore, it is a context about which there is a grave paucity of process knowledge (Eckhardt et al., 2006; Pandya, 2009), in particular on individual interventions (Murphy and Meis, 2008; Sheehan et al., 2012, though see Lømo et al., 2016, 2019). We argue that our mode of inquiry can provide meaningful insights to the field by providing a “distinctive form of practical knowledge that uniquely captures the complexity of naturally-occurring phenomena” (McLeod, 2013, p. 382; Tebes, 2000; Barlow and Nock, 2009; Dattilio et al., 2010).

Fourth, our sample consisted of men only. Whilst this is not unusual in the context of intimate partner violence, it might limit the usefulness of our findings for psychotherapy with women who use IPV. There might be different patterns or reasons for the use of violence among women (Hamberger, 1997; Hamberger and Guse, 2002). However, we believe that the perspective we offer on ambivalence can be useful across both genders and various client groups.

Finally, a note on whether this analysis can be trusted and believed (Morrow, 2005). We view validation as inherent in the research process (Kvale, 1989; Charmaz, 2013b), and have sought to strengthen validity through a continuous process of contrasting and comparing: between the authors’ different interpretations, between emerging categories and the empirical material and between our interpretations and the existing literature. Furthermore, we have sought to be transparent in our descriptions of the steps and choices in the analysis and give ample examples of our interpretations from the empirical material, in order to allow the reader to assess methodological integrity (Levitt et al., 2018). We were also able to use multiple sources and qualities of outcome measures to ensure what we assessed a fitting context for our inquiry: therapies characterized by s strong and enduring working alliance and a good outcome. Research on violence-focused interventions has weaknesses here, for instance by often relying uniquely on reconviction data or perpetrator self-report. In both cases, one may assume that some violence goes under the radar, as demonstrated by studies finding a discrepancy between client- and partner reporting in general (Armstrong et al., 2002). That discrepancy is present in the ATVT-sample as well, though on a much smaller scale (Strandmoen et al., 2016). In combination with our theoretical and clinical arguments for understanding ambivalence as a part of the change process, this should hopefully allow the readers to assess whether our findings are useful for them to “explore and test in their own way and in their own contexts” (Reichelt, 1995, p. 142, author’s translation).

### Implications for Further Research

The gender perspective is touched upon in this analysis, but future work could usefully be situated more broadly than in the immediate therapeutic context. A broader gendered analysis of this material with regards to how notions of masculinity may hinder various phases of the change process (seeking help, therapeutic work, implementing change in real life) or how the therapy changes (or doesn’t change) gendered power relations in the relationship could be relevant and useful (Haavind, 2000).

Another interesting avenue is to explore the therapeutic interactions around client ambivalence. Such an analysis could for instance be a conversation analysis (Drew, 2015), a dialogical discourse microanalysis (Martinez et al., 2012) or a dialogical sequence analysis (Leiman and Stiles, 2001; Leiman, 2012). Furthermore, we observed that these categories of ambivalence occurred at different timings and frequencies throughout the therapies. A more detailed analysis of the development of ambivalence in form and content throughout the change process could be useful. Finally, it could be very enlightening indeed to ask both client and therapist how they experience the phenomenon of ambivalence in therapy. After all, experiential correspondence is an important part of the truth (Stiles, 1981). This could be done with Interpersonal Recall (Rennie, 1994) and form a foundation for user involvement in further analysis.

## Concluding Remarks

When facing an issue with such devastating consequences as intimate partner violence, the clinician might feel a sense of urgency. The nature of the actions being described could even provoke resistance and hostility in him or her. As understandable as this is, there is a risk of creating tension with the client’s need to take time to overcome shame, to be seen and to see himself as a good person too, and to work with the deadlocked patterns of thoughts and emotions that maintain his violence. We argue that client ambivalence as we have described it can be understood as an opportunity for change and perhaps also one of the core mechanisms that has made it difficult for the client to break out of his patterns of violence on his own. From this perspective, we see a client that has done and is doing something bad, but also actively engages in his own process of change by building the therapeutic alliance, showing the therapist what he needs help to co-create a new understanding of and braving the vulnerability and insecurity inherent in this process. This understanding can help the clinician listen for ambivalence and understand it as client agency and openness to change, and thus get in a better position to help. We hope therefore that this article makes a useful contribution to building clinically relevant knowledge regarding how we might help these clients, so that we can reduce the suffering of not being able to go home, close the door on the world, and feel safe.

## Ethics Statement

This study was carried out in accordance with the recommendations of General guidelines for research ethics, Norwegian Regional committee for medical and health research ethics (REC) with written informed consent from all subjects. All subjects gave written informed consent in accordance with the Declaration of Helsinki. The protocol was approved by the Norwegian Regional committee for medical and health research ethics (REC).

## Author Contributions

MT-K took the lead in the creative and analytic work, decided the research questions and design, and selected the meaning units and suggested categories. BL and OT contributed in the analysis, by discussing examples of ambivalence and in developing the categories. This was done in regular meetings with all authors and in meetings MT had with OT and BL separately. MT wrote the manuscript, with OT and BL giving feedback on and suggestions for the text both separately and in conjoined meetings.

## Conflict of Interest Statement

The authors declare that the research was conducted in the absence of any commercial or financial relationships that could be construed as a potential conflict of interest.
